# Supplementary figures and images for: The stem rust fungus Puccinia graminis f. sp. tritici induces centromeric small RNAs during late infection that are associated with genome-wide DNA methylation
Source: BMC Biol. 2021 Sep 15;19:203. doi: 10.1186/s12915-021-01123-z (PMC8444563; doi:10.1186/s12915-021-01123-z)

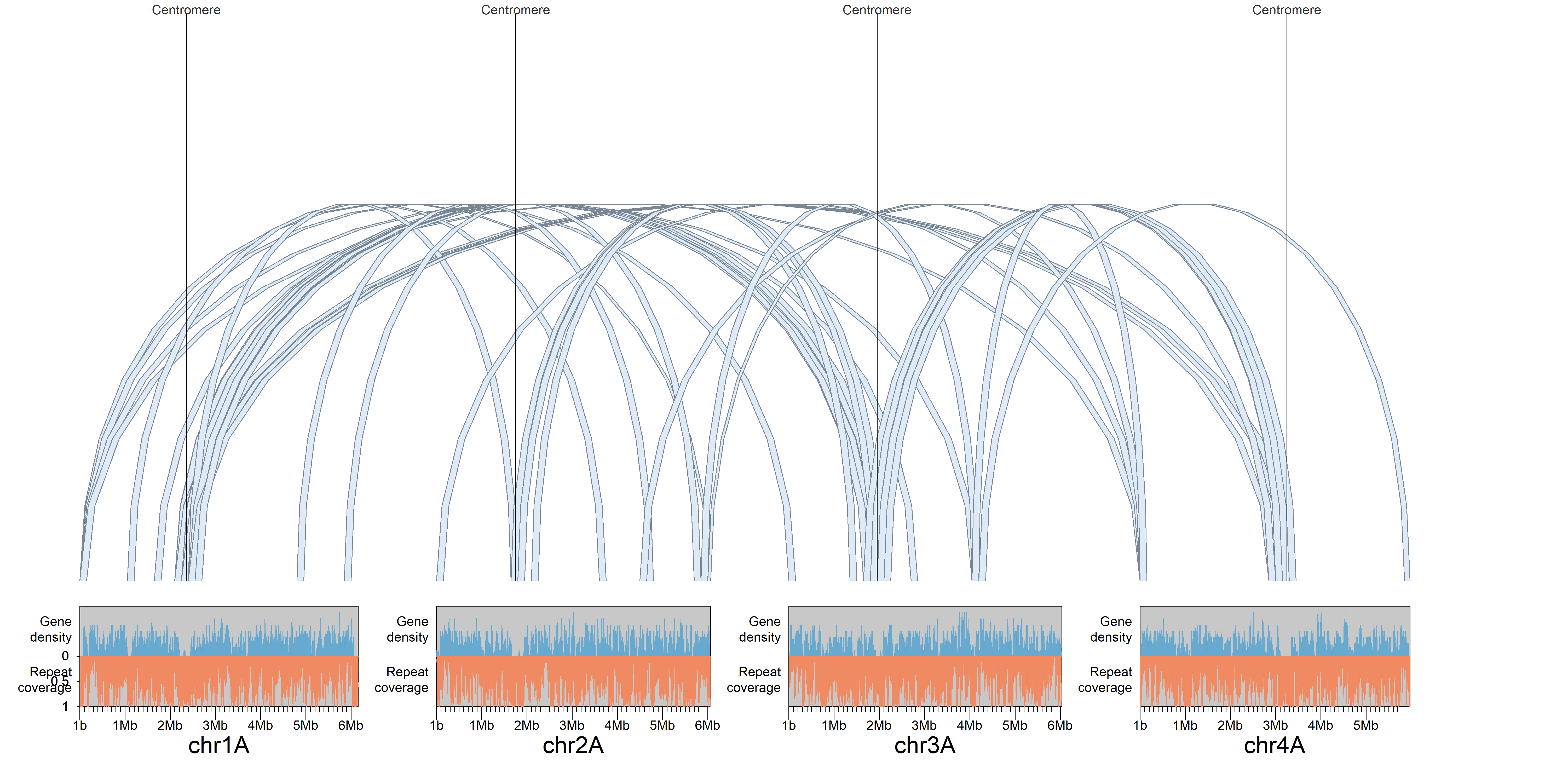

Supplement: Supplementary file 3 — Additional file 3: Fig. S3. 150 kbp bins with interaction frequency > 5 in the Hi-C interaction matrix are shown between chromosomes 1A, 2A, 3A and 4A. The putative centromeric regions share strong connections with each other. Densities of expressed genes and coverage of repetitive elements are shown with window size 10 kbp. The centromeric regions are gene-poor regions with high repetitive element coverage. [file 12915_2021_1123_MOESM3_ESM.png]

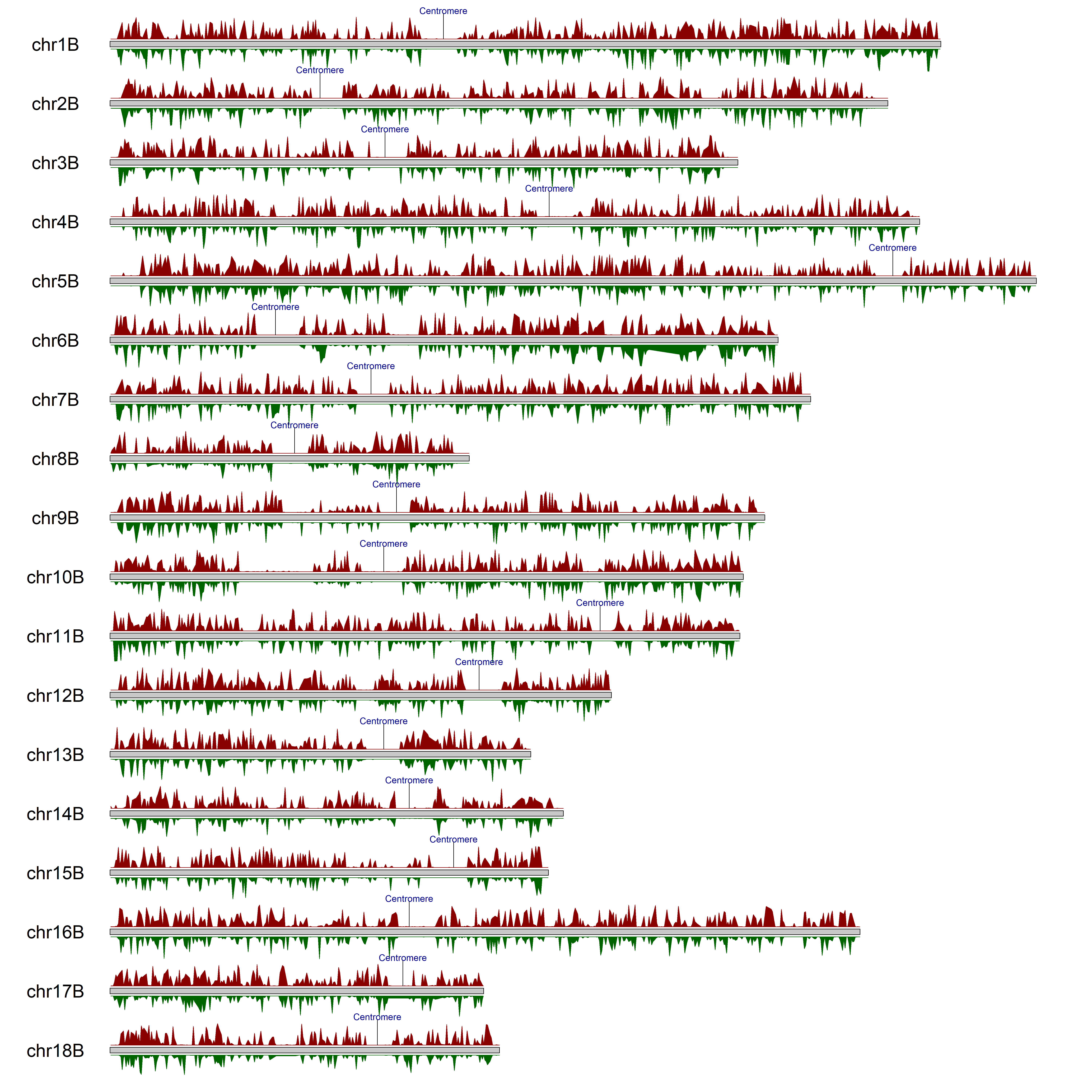

Supplement: Supplementary file 4 — Additional file 4: Fig. S4. The positions of the centromeres in haplotype B as indicated by the Hi-C contact map are in transcriptionally silent genomic regions. Reads per million (RPM) for the late infection (7 dpi) and germinated spores RNAseq samples are shown in red and green, respectively (10 kb windows, RPM from 0-100 are shown for clarity). [file 12915_2021_1123_MOESM4_ESM.png]

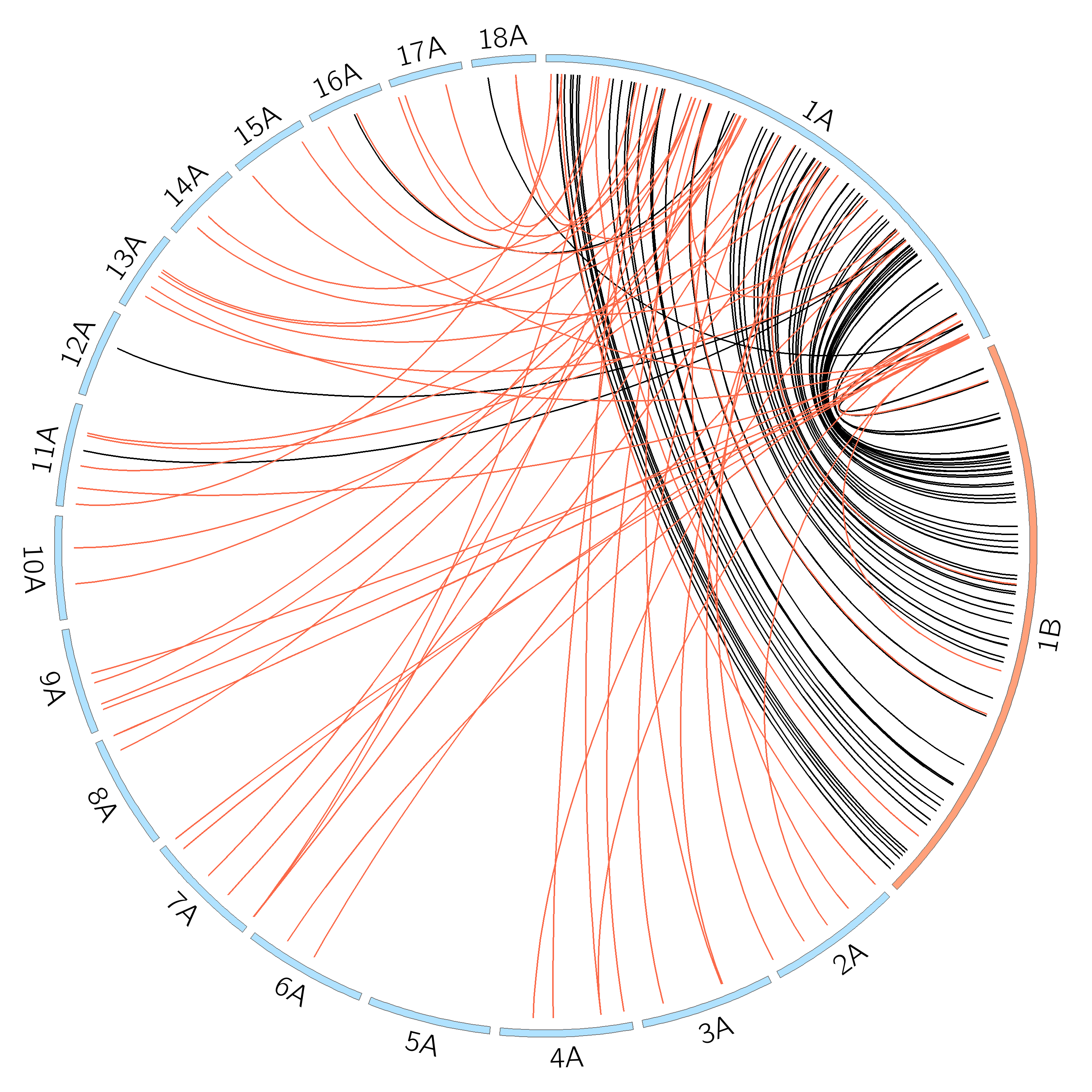

Supplement: Supplementary file 14 — Additional file 14: Fig. S14.Pgt allelic sRNA pairs and their genomic localization for chromosome 1A. Pgt sRNAs that are up-regulated in germinated spores (late infection) and their homologous counterparts are shown with black (red) links. sRNAs that are up-regulated in germinated spores appear to be in syntenic on the two haplotype chromosomes 1A and 1B (shown at twice their size, other chromosomes shown at 0.2 their size). In contrast, sRNAs that are up-regulated during late infection on chromosome 1A have homologous counterparts on all other chromosomes except 5A and 12A. [file 12915_2021_1123_MOESM14_ESM.png]
