# Supplementary material for: The stem rust fungus Puccinia graminis f. sp. tritici induces centromeric small RNAs during late infection that are associated with genome-wide DNA methylation
Source: BMC Biol. 2021 Sep 15;19:203. doi: 10.1186/s12915-021-01123-z (PMC8444563; doi:10.1186/s12915-021-01123-z)
Supplement: Supplementary file 15 — Additional file 15. Original blots. [file 12915_2021_1123_MOESM15_ESM.pptx]

## Slide 1
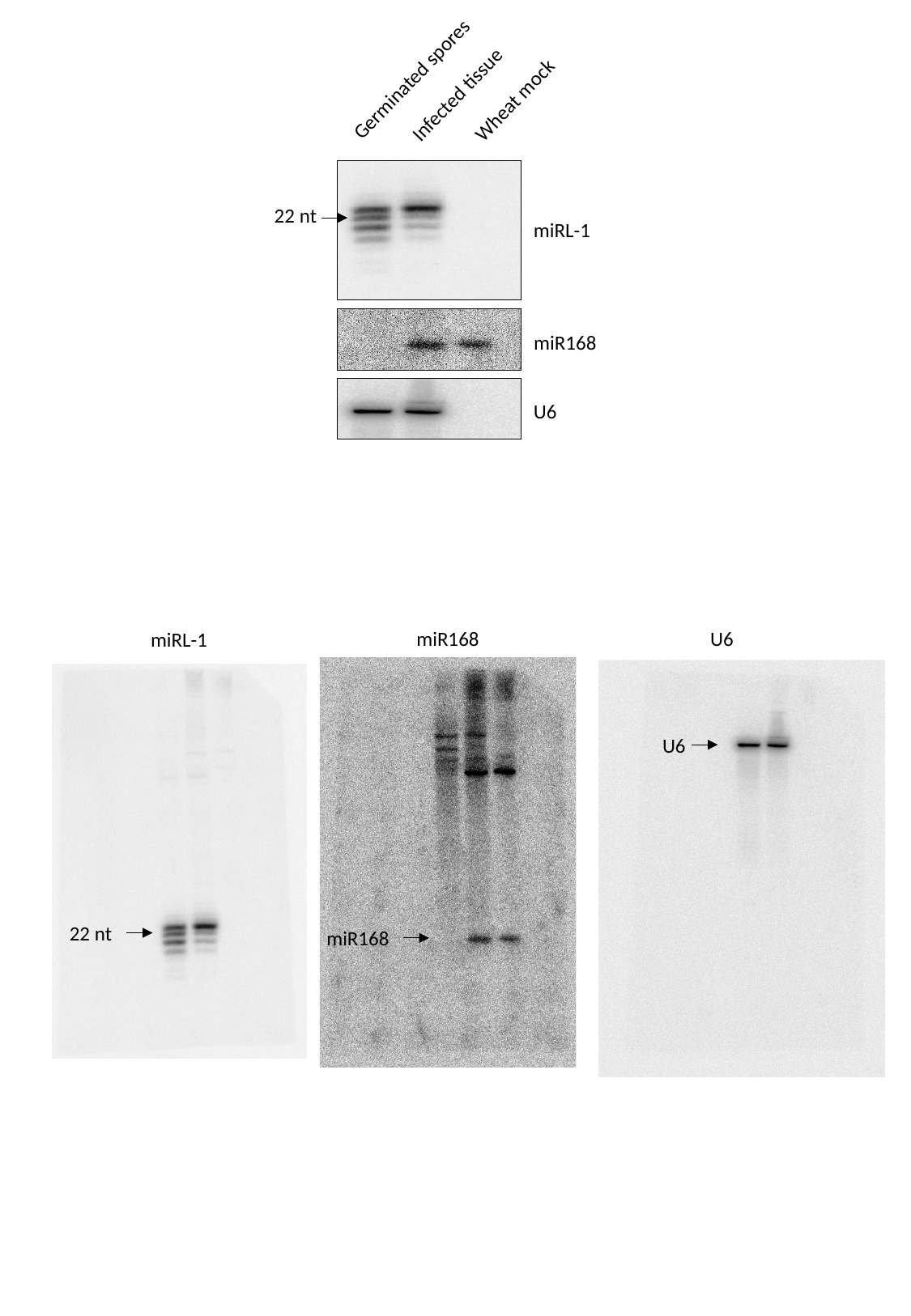

Germinated spores
Infected tissue
Wheat mock
22 nt
miRL-1
miR168
U6
miR168
U6
miRL-1
U6
22 nt
miR168

## Slide 2
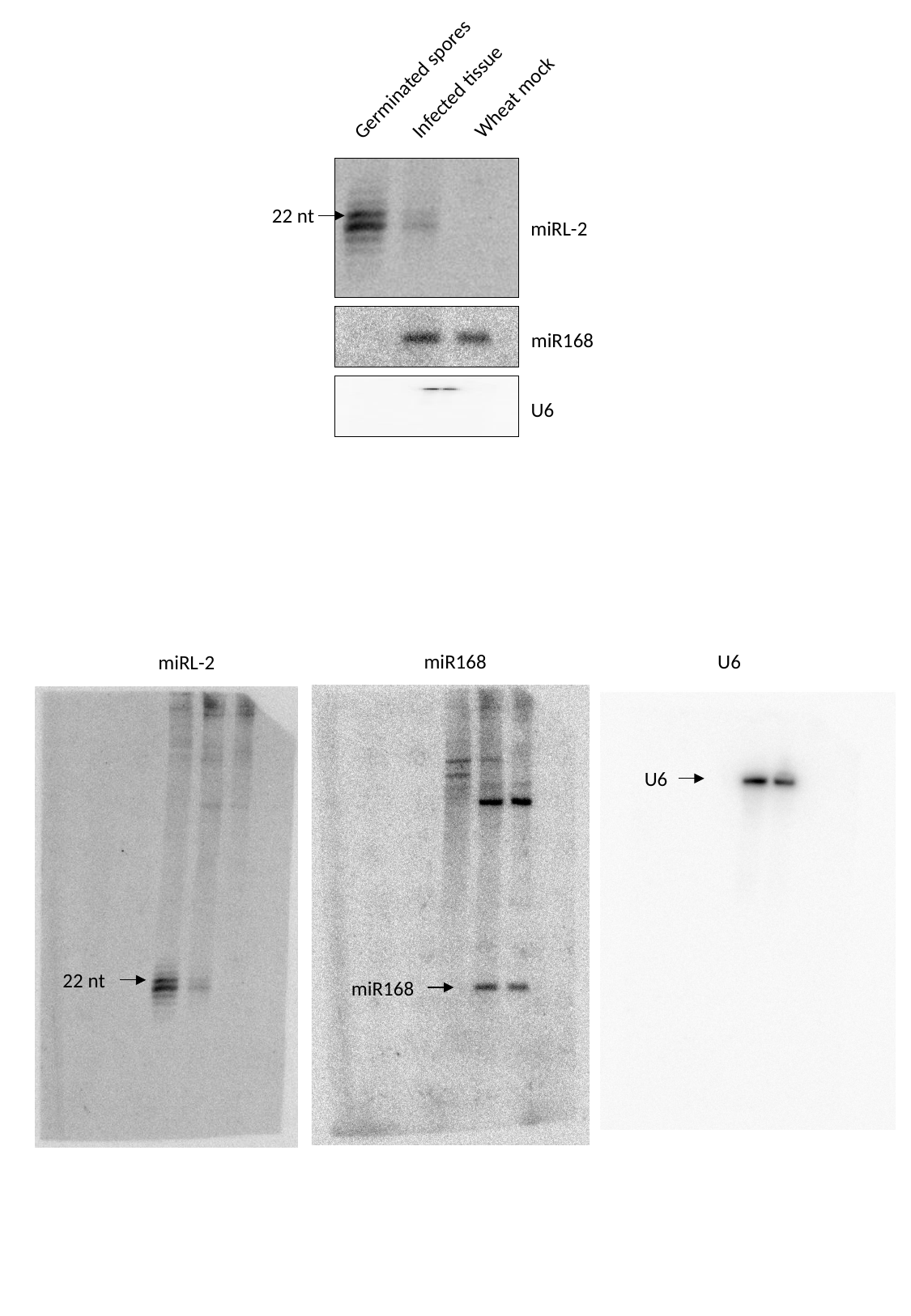

Germinated spores
Infected tissue
Wheat mock
22 nt
miRL-2
miR168
U6
miR168
U6
miRL-2
U6
22 nt
miR168
